# Supplementary material for: Ending the HIV epidemic using National HIV Behavioral Surveillance (NHBS): Recommendations based on DC model
Source: PLoS One. 2021 Jul 22;16(7):e0253594. doi: 10.1371/journal.pone.0253594 (PMC8297872; doi:10.1371/journal.pone.0253594)
Supplement: S1 Table — (DOCX) [file pone.0253594.s001.docx]

Supplement Tables 1. Characteristics of networks

‘

Supplement table 1. Cont’d

Supplement table 1. Cont’d
